# Do hospitalisation rates increase following discharge from an Early Intervention for Psychosis service? A naturalistic cohort study

**DOI:** 10.1192/j.eurpsy.2026.10531

**Published:** 2026-07-31

**Authors:** J. Evans, G. Kavanagh, A. Connolly, S. Arora, M. Pereira, R. Whale

**Affiliations:** 1 Sussex Partnership Foundation Trust; 2 Brighton and Sussex Medical School, Brighton, United Kingdom; 3 Faculty of Psychology and Educational Sciences, University of Coimbra, Coimbra, Portugal

## Abstract

**Introduction:**

Contention remains as to the impact of withdrawal of Early Intervention Services (EIS) on subsequent hospitalisation rates. The UK NICE guideline continues to state that such clinical gains of EIS are diminished following discharge and recommends further study at 5 years (accessed July 2025; https://www.nice.org.uk/Guidance/CG178).

**Objectives:**

We aimed to explore hospitalisation rate by year in the UK Sussex First Episode Psychosis Cohort study (S1P).

**Methods:**

We retrospectively recorded hospitalisations in the S1P cohort for those who had continued to receive secondary mental health care at 5 years from first psychotic episode, including 2 years following discharge from EIS. Data was collected between 2009 and 2017. Ethical conduct of the study was approved by the Sussex Partnership NHS Foundation Trust.

**Results:**

184 patients received 5 years of care following a first episode of psychosis (52% of the original cohort enrolling with EIS). 69% had at least 1 hospitalisation episode between baseline and 5 years. Actual total new hospitalisations per year were 72 at baseline, then 59, 44, 49, 54 and 41 in subsequent years. Mean hospitalisations per person per year excluding the index admission when comparing the 0-3 (under EIS care) vs 4-5 years period was **0**.**275** [95% CI - 0.215 to 0.335] vs **0.219** [0.159 to 0.278] respectively (**n = 183**; p = 0.050).

No changes in external factors influencing overall bed availability were identified during the study period

**Conclusions:**

The group of patients requiring care up to 5 years had initial declining hospitalisation rates but relative stability following the first year. The group not requiring 5 years of specialist mental health care, not studied here, likely had much lower hospitalisation. The hypothesis of rebound hospitalisation in the 2 years following EIS discharge is not supported.

**Disclosure of Interest:**

None Declared

